# The Boring Billion, a slingshot for Complex Life on Earth

**DOI:** 10.1038/s41598-018-22695-x

**Published:** 2018-03-13

**Authors:** Indrani Mukherjee, Ross R. Large, Ross Corkrey, Leonid V. Danyushevsky

**Affiliations:** 1 Centre for Ore Deposits and Earth Sciences (CODES), University of Tasmania, Hobart, Australia; 2 Tasmanian Institute for Agricultural Research (TIA), University of Tasmania, Hobart, Australia

## Abstract

The period 1800 to 800 Ma (“Boring Billion”) is believed to mark a delay in the evolution of complex life, primarily due to low levels of oxygen in the atmosphere. Earlier studies highlight the remarkably flat C, Cr isotopes and low trace element trends during the so-called stasis, caused by prolonged nutrient, climatic, atmospheric and tectonic stability. In contrast, we suggest a first-order variability of bio-essential trace element availability in the oceans by combining systematic sampling of the Proterozoic rock record with sensitive geochemical analyses of marine pyrite by LA-ICP-MS technique. We also recall that several critical biological evolutionary events, such as the appearance of eukaryotes, origin of multicellularity & sexual reproduction, and the first major diversification of eukaryotes (crown group) occurred during this period. Therefore, it appears possible that the period of low nutrient trace elements (1800–1400 Ma) caused evolutionary pressures which became an essential trigger for promoting biological innovations in the eukaryotic domain. Later periods of stress-free conditions, with relatively high nutrient trace element concentration, facilitated diversification. We propose that the “Boring Billion” was a period of sequential stepwise evolution and diversification of complex eukaryotes, triggering evolutionary pathways that made possible the later rise of micro-metazoans and their macroscopic counterparts.

## Introduction

The “Boring Billion” (period between 1800 and 800 Ma) in Earth’s history, also referred to as the “dullest period in Earth’s history”^1^ is believed to represent a period of geobiological stasis^2^. The so-called stasis is manifested as a remarkably stable and flat carbon isotope trend due to prolonged lack of nutrient supply and tectonic stability^2^. Other stable and undisturbed trends are depicted by S, Mo, Cr, Sr isotopes, and more particularly, by low values of trace element concentrations and P in marine black shales^3–10^. Outcomes of previous studies point towards a delay in the evolution of complex life primarily due to lack of oxygen in the atmosphere^11^. It is believed it was not until the Neoproterozoic (towards the end of the Boring Billion), that a great diversity in complex life (both microscopic and macroscopic) developed. Absence of banded iron formations, evaporites, phosphorites, glaciation events and major ore deposits related to convergent plate margins (Orogenic Au, porphyry, VHMS and MVT deposits) also correlate with the period of stasis^12^. Plate motions at that time were suppressed, possibly owing to stagnant lid tectonics, with modern plate tectonics not operative until towards the end of the Neoproterozoic^13^. General consensus therefore is that the 1800–800 Ma period represents a billion years of geological standstill that stalled evolution of complex life.

Contrary to above, we have identified certain gaps in the understanding of the “Boring Billion” period. *First*, even though geochemical proxies indicate “stasis”, certain key evolutionary breakthroughs occurred during this time span. These include the appearance of eukaryotes (including its cell organelles) possibly via endosymbiosis, multicellularity and origin of sexual reproduction^14–18^, and the evolution of precursors of metazoans (Urmetazoa)^19^. Without these developments, any subsequent metazoan and macroevolution would be impossible. *Second*, we observe the first major diversification of eukaryotes (Crown group)^20–22^, the appearance of metaphytes^15,18^ and metazoans^15,23^, between the Great Oxidation Event (GOE) and the Neoproterozoic Oxidation Event indicating that oxygen may not have been the only driver of these developments. Experiments also support low oxygen requirements of modern analogues of primitive metazoans^24^. The sudden appearance of metazoans with no obvious prior evolutionary developments is hard to comprehend. That is because metazoan evolution requires multicellularity, development of scaled epithilea and extracellular digestion, a nervous system, mesoderm, bilaterity and establishment of a tubular gut^25^. Also, any one element (e.g. O) cannot account for the course of biologic evolution^26^. A suite of bio-essential elements including H, C, N, O, P, S, Cl, Na, Mg, K, Ca, Al, Si (macro elements) and Fe, V, Cr, Mn, Co, Ni, Cu, Zn, Mo, B, F, As, Se (trace elements) are required by all kinds of life forms^26^.

Interestingly, bio-essential trace element abundance, unlike major elements, does not necessarily signify its importance in an organism as it is utilized only if it has functional roles^26^. Scarcity of an element plays an equally important role as it encourages organisms to adapt to an alternative element that has similar functional roles. This results in the selection of new protein molecules with advanced roles^27,28^. Thus, knowledge of multi-trace element variability in the ocean, particularly multi-trace element minima, is an essential aspect in understanding biologic evolution that has not been previously addressed. It has been noted by Sterner^29^ and Morel *et al*.^30^ that the major elements have received far more attention than the trace elements as potential limiting nutrients related to biological evolution.

The present study focuses on using a technique where trace element concentrations in sedimentary pyrite, determined using LA-ICP-MS, provide useful first order insights on trace element availability (abundance and scarcity) in the ocean with time^8^. Without undermining the role of macronutrients (such as P, N, O, C etc.) we aim to establish the link between trace element availability and biologic evolution through the Boring Billion period.

## Method and Materials

Our method involves measuring trace element concentrations in sedimentary pyrite in black shales using Laser Ablation- Inductively Coupled- Mass Spectrometry (LA-ICP-MS) as proxies for atmospheric oxygenation and nutrient trace element availability^8^ (supplementary information: SI). It is based on the premise that the availability of most trace elements in seawater is controlled by a combination of oxidative weathering on land, and erosion/run-off to the ocean related to active tectonics. Subsequently, the trace elements become absorbed into sedimentary pyrite forming in organic matter-bearing muds depositing in anoxic portions of the ocean. Sedimentary pyrite analyses are preferred to whole rock shale analyses as the method is more sensitive to trace element concentrations, and trace elements are preferentially partitioned and concentrated into pyrite compared to whole rock^31,32^. The validity of the technique has been the subject of several publications^33–36^ which includes the proof of concept paper^8^. Additional information is also provided in the SI.

The study provides new analyses from the Riversleigh Siltstone in the Mt. Isa Basin in Australia and collates data from ~40 marine, least metamorphosed (lower greenschist and below), undeformed organic matter rich-sedimentary black shale formations from various sedimentary basins around the world (Table SI1a,b)^8,36,37^. Samples were mainly collected in the form of drill cores in order to ensure the pyrite was well preserved and not oxidised. Polished laser mounts were prepared for petrological analysis using reflected light microscope followed by LA-ICP-MS pyrite analyses for trace elements at CODES, University of Tasmania^8^. Polished mounts were studied under reflected light in order to select samples that contain fine grained early-formed sedimentary pyrite and discard coarse, recrystallised and diagenetically altered pyrite. Most samples comprised sedimentary pyrite in the form of individual microcrystals (5 to 10 u), aggregates of microcrystals, framboids and nodular concretions in black shales. Samples containing coarser euhedral pyrites were discarded as trace element budget is affected due to recrystallization^8,31,32,34^. Analyses were carried out using CompexPro 110 ArF Excimer laser microprobe equipped with S155 cell and coupled to an Agilent 7700 ICP-MS. A total of ~ 1400 robust analyses were obtained after exclusion of analyses that did not meet the criteria outlined for sedimentary pyrite (done on the basis of trace element ratios such as Ni/Co >1 in pyrite)^34^. Standards (or reference materials) were run at the start, about after every two samples (about every 1.5 hours) and at the end, in order to calculate the drift of the instrument (see SI)^8^. The signal from the mass spectrometer was generated in counts per second (cps) and was processed by CODES in-house software designed according to standard methods^38^ (Longerich *et al*.), using Fe as the internal standard element. Additional information on LA-ICP-MS data processing is provided in the SI.

### Statistical methods

**Figure 1:**
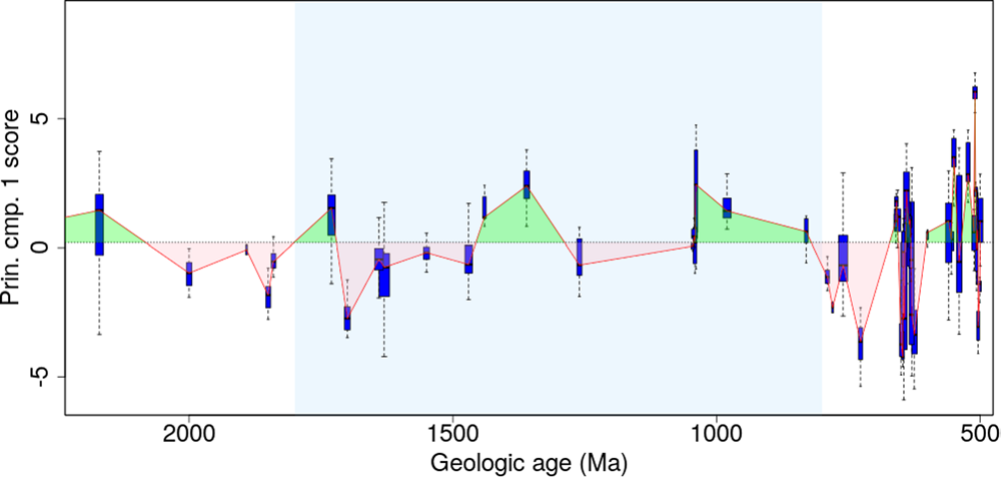
First principal component scores using all trace element variables against geologic age. Shown are the PCA scores for the first dimension as boxplots and also the median trend as a solid line. The overall median score is shown as a dotted line with areas above it coloured green, and below coloured pink.

**Figure 2:**
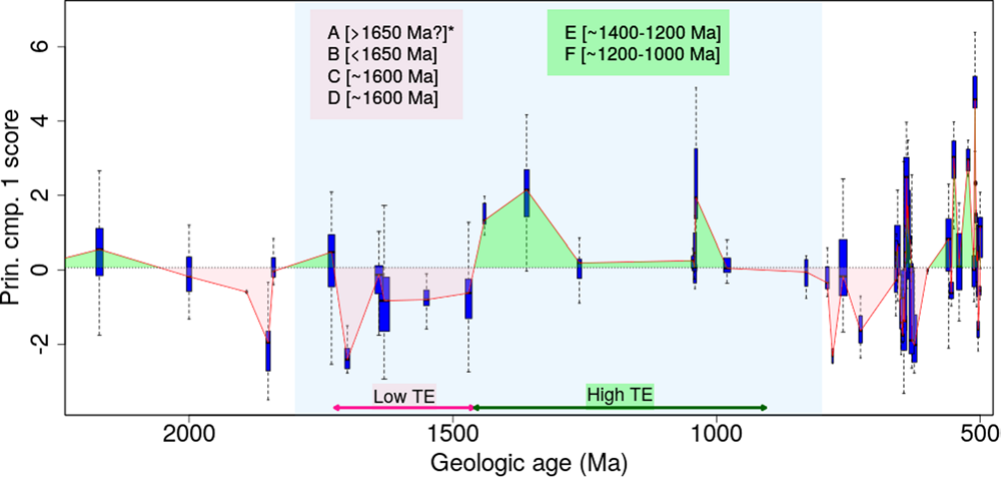
First principal component scores for selected trace element (TE) variables (Se, Ni, Co, Zn, Mo, Cd) against geologic age. Shown are the PCA scores of the first dimension as boxplots and also the median trend as a solid line. The overall median score is shown as a dotted line with areas above it coloured green and below coloured pink. (**A**) Endosymbiosis; (**B**) First eukaryotes and its cell organelles^14–16^; (**C**) Multicellularity^18^; (**D**) Sexual reproduction^18,41^; (**E**) Diversification and radiation of eukaryotes^20–22,53–59^; (**F**) Crown group eukaryotes^15^. *Exact timing of endosymbiosis is unknown but must have occurred prior to the appearance of first eukaryotes.

The trace element data were standardised by subtracting the mean and dividing by the standard deviation and then analysed using principal components analysis (PCA); (Table SI2) (PCA)^39^. The standardisation was to ensure that the results were not influenced unduely by variables of large magnitude. The PCA was calculated using PROC PRINCOMP in the SAS System version 9.3. The scores from the first component of the PCA were used to construct boxplots that were plotted against the corresponding geologic age. The PCA scores of the first component were plotted against geologic age for all trace element data in Fig. 1 and for selected variables such as Ni, Co, Se, Zn, Mo, Cd in Fig. 2. In both figures, the scores are presented as box plots along with the median trend (solid line) and the overall median score (dashed line). The use of boxplots allowed the spread of scores at each age to be represented graphically. Boxplots were calculated using R version 3.3.3.

### Data availability

All data generated or analysed during this study are included in this published article (and its Supplementary Information files)

## Results

The pyrite-LA-ICP-MS analyses for a suite of trace element (Co, Ni, Zn, Se, Mo, Cd, Cu, Mn, As, Ag, Sb, Tl and Pb) are presented in Table SI1(Supplementary Information). The data are a compilation from Large *et al*.^8,36,37^ along with additional new analyses from this study. The first component of the PCA accounted for 31% variation in the data using all trace element variables, and 38% when using the selected variables (Ni, Co, Se, Mo, Zn and Cd) (Table 1). The eigenvector for the first component of the PCA using all trace element variables and for selected variables, had positive coefficients (Table 1).

**Table 1:** Shown are the PCA eigenvector coefficients and eigenvalues when using all variables or using the selected variables.

Analysis	Variable	Prin1	Prin2	Prin3
	Co	0.087	0.560	0.015
	Ni	0.247	0.498	−0.037
	Zn	0.294	−0.202	0.396
	Se	0.304	0.056	−0.102
	Mo	0.330	−0.218	−0.045
	Cd	0.297	−0.146	0.235
	Cu	0.294	0.228	0.266
All variables	Mn	0.209	0.008	0.495
	As	0.270	−0.063	−0.531
	Ag	0.371	−0.031	−0.073
	Sb	0.349	0.095	−0.394
	Tl	0.135	−0.505	−0.119
	Pb	0.273	−0.073	0.035
	Eigenvalue	4.07	1.94	1.53
	% explained	31	15	12
	Co	0.070	0.702	0.388
	Ni	0.337	0.621	−0.131
Selected variables	Zn	0.466	−0.227	0.569
	Se	0.466	0.039	−0.651
	Mo	0.451	−0.132	−0.152
	Cd	0.494	−0.227	0.250
	Eigenvalue	2.25	1.54	0.861
	% explained	38	26	14

The interpretation of the first component is that it corresponds to a weighted average of the trace element variables. In other words, the first component of the PCAs provided a proxy for the trace element concentrations. In Fig. 1, the trace element trend is mostly below the median value between 2500–1400 Ma (except at 2500, ~1900 and 1730 Ma). Between 1400–800 Ma, the trend is above the median value except at ~1260 Ma. In Fig. 2, where only selected trace elements have been used to derive the principal component score, we observe a similar trend as Fig. 1. The trace element trend is initially above the median value but drops below it and remains so until 1400 Ma (Fig. 2). Between 1400–800 Ma the trace element trend is above the median value.

During the period 2000–1400 Ma there were 4 biological innovations (Eukarya, organelles, multicellularity, sex) discussed below. We can calculate the probability of the 4 events occurring in this period by assuming that these 4 events could occur independently and with uniform probability anytime between 2000 and 800 Ma. Since the periods 2000–1400 Ma and 1400–800 Ma are equal length intervals and, by our assumption, the events could occur with the same probability in each, then the probability of them co-occurring *only* between 2000–1400 Ma would be (1/2)^4^ = 0.0625.

## Discussion

Here, we interpret the trace element trend in light of biologic evolution observed in the paleontological rock record in the Boring Billion period. Figures 1 and 2 demonstrate that trace element concentrations were mostly below the median value between 2000 and 1400 Ma. This is in agreement with previous research where low trace element concentrations have been proposed for this particular time span^3,4,6,9^. This can be attributed to the drop in oxygen levels after the GOE event. As evident from various geochemical proxies in the rock record, the GOE was transient in nature and oxygen dropped to lower levels at ~2000 Ma^8,11,40^. This is also clear from the Se/Co in pyrite (oxygenation proxy; see SI for more information) trend through time in this study (Fig. SI1). The decrease in bio-essential trace element concentrations (Ni, Co, Se, Zn, Mo, Cd) is possibly due to a decreased trace element flux in the ocean as a result of decrease in oxidative weathering (Figs 1, 2). Coinciding with this drop in oxygen and trace element concentrations, a number of key evolutionary breakthroughs are believed to have occurred, including: appearance of first eukaryotes, acquisition of certain cell organelles (or cell components such as plastids, mitochondria, nucleus, endoplasmic reticulum, cytoskeleton etc.) and multicellularity between 2000 and 1400 Ma^14–16^. Based on the paleontological record, the origin of sexual reproduction is also believed to have evolved between 2000–1400 Ma^18,41^. The micro-paleontological record confirms that the phenomena were global in nature as proposed by previous studies; for instance, in the Paleoproterozoic Changcheng and Ruyang Groups, China^42–44^, Vindhyan Supergroup, India^45^, and Roper Group, Australia^46^, and Mesoproterozoic of Jixian Group (1.56 Ga), China^47^; Belt Group (1.5 Ga), USA^48^; Kotuikan Fm (~1.5 Ga) Russia^49^; Roper Group (1.5 Ga), Australia^22^. Interestingly, most of the key biological innovations took place in a period where bio-essential trace element concentrations are consistently low between 2000 to 1400 Ma. These particular events were not all independent of each other in that Eukarya had to evolve before organelles (although some prokaryotes possess analogous structures)^50^. However, the 4 events, Eukarya, acquisition of organelles, sex, and multicellularity, could be considered plausibly to have happened in any order and time, although the latter two may not be independent^17^. If they are independent events then the likelihood of their co-occurrence during the low trace element period has been demonstrated to be quite small (~0.0625).

Unlike past studies, the trace element trend observed in the total Boring Billion period is not entirely low and flat, but after 1400 Ma, is, followed by a long period of relatively higher trace element concentrations (1400–800 Ma); i.e., higher than the median value for the Proterozoic. We observe an increase in trace element concentrations at around 1400 Ma which also coincides with an oxygenation event recognized using independent geochemical proxies (pyrite redox-sensitive trace element chemistry in black shales^31^, Mo isotopes in black shales^51^ and U isotopes^52^). Interestingly, this event is coeval with a major diversification of eukaryotes^20–22^. Complex cellular morphological capacities (cytoskeletal architecture) were observed and eukaryote habitation spread to a wider range of environments^20,21^. Diversification of eukaryotes is also noted in the Kamo Gp (1.3 Ga)^53^; Kaltasy Fm (1.4–1.45 Ga)^54^; Sarda Fm (~1.3 Ga), India^55^; Arctic Canada (~1.3 Ga)^56,57^; in the 1.1–0.9 Ga Mbuyi-Mayi Supergroup, DR Congo^58^; in the 1.1 Ga Atar/El Mreiti Gp, Mauritania^59^. This was followed by the diversification of crown group eukaryotes between 1200–1100 Ma^15^ and the emergence of a gene (1000–800 Ma), recently identified, that is linked with protein and choline kinases responsible for cell adhesion and transfer of signals within cells efficiently^60,61^. The period ended with the appearance of metazoans at ~750 Ma^15,23^ and the origin of fungi between 760 and 1000 Ma^62^.

In summary, key biological innovations in eukaryotes seemed to have co-occurred during the low trace element period, followed by a broad-scale diversification of eukaryotes, in the relatively high trace element period. We attribute this trend from a prokaryotic community (>1800 Ma) to the formation of the first eukaryotes (1800–1500 Ma) and their diversification (1400–800 Ma) to nutrient trace element availability as shown in Figs 1 and 2. Of course, macronutrient availability too may have played a critical role. However, previous work suggests a low and stable P and N through most of Earth’s history until towards the end of the Boring Billion (~800 Ma)^10^. This further supports the putative role of trace elements in shaping the course of evolution.

We propose that co-limitation of nutrient trace elements may have caused an environmental stress, a plausible driver of the biological innovations in the early part of the Boring Billion including endosymbiosis^63^, whereby unicellular prokaryotes are forced into a symbiotic relationship, due to nutrient limitation, conceivably resulting in Eukarya. Hoffmann and Hercus^64^ have previously proposed environmental stress as a major driver for evolutionary changes (DNA/genotypic/phenotypic variations, adaptation capacities). A decrease in the population (clearing of the ecological space), as a result of stressful conditions, causes renewed predation and competition that promotes subsequent evolutionary radiations in the newly reconstructed ecological space^64,65^. Another possible mechanism for evolutionary change are major population bottleneck events, such as may be expected during low trace element stress periods, which were then fixed as a result of small population sizes^50^. Experiments have demonstrated that stress can double rates of stress-induced genetic mutations in bacteria whereby new genes are created with different, and often advanced, functions^66^. We propose that a prolonged period of low nutrient trace element conditions may have contributed to an environmental stress that triggered the key biological innovations we observe in the rock record.

On the other hand, an increase in nutrient trace elements (after a prolonged nutrient crisis) may have facilitated diversification of the eukaryotes between 1400 and 800 Ma. Once the diversification process commenced, the eukaryotic community expanded in the marine realm. One could pose the question that if nutrient conditions did in fact improve, what delayed the rise of animals until ~750 Ma? It is quite possible that the evolution and expansion of complex eukaryotic communities played a significant role in fostering ocean oxygenation (as proposed by Lenton *et al*.)^67^ and bring about major changes in P and N cycles making the conditions conducive for the subsequent evolution of metazoans and their macroscopic counterparts. Therefore, the Boring Billion period is a critical time in Earth’s evolutionary history when the prerequisites for macroevolution were established.

## Conclusions

Our primary conclusions lead to a paradigm shift in understanding evolution during the Proterozoic. *First*, the Proterozoic ocean witnessed periods of both low and high trace element availability; trace element trends are not flat and uniform as previously assumed. *Second*, we emphasize that the low nutrient trace element periods were possibly essential triggers in the course of evolution. Previous studies claim low concentrations of trace element may have stalled evolution^3^. However, we argue that these periods of trace element crisis forced organisms to explore their options to adapt to stressful conditions and promoted mechanisms to cope and evolve. On the upside of nutrient trace element cycles, when conditions improved in terms of high trace element availability from 1400 Ma to 800 Ma, organisms diversified. Thus, the need for both unfavorable and favorable nutrient conditions may have been required to generate the necessary evolutionary pressure and diversification respectively through time. *Third*, we propose that the high and low trace element periods in the “Boring Billion” may have played a critical role in establishing the prerequisites for metazoan evolution. It is unlikely that metazoans appeared all at once without prior evolutionary achievements. Therefore, sequential stepwise evolution and diversification of complex eukaryotes was likely a result of fluctuating nutrient trace element conditions (stress/stress-free) through the “Boring Billion”.

## Electronic supplementary material

Supplementary information

Dataset 2

Dataset 1
