# Supplementary material for: The Boring Billion, a slingshot for Complex Life on Earth
Source: Sci Rep. 2018 Mar 13;8:4432. doi: 10.1038/s41598-018-22695-x (PMC5849639; doi:10.1038/s41598-018-22695-x)
Supplement: Supplementary file 1 — Supplementary information [file 41598_2018_22695_MOESM1_ESM.pdf]

The Boring Billion, a slingshot to complex life on Earth

Indrani Mukherjee, Ross Large, Ross Corkrey and Leonid Danyushevsky

University of Tasmania, Hobart, Australia

1. Methods and materials

1.1 Using sedimentary pyrite trace element

Certain redox-sensitive trace elements (Mo, U, Cr, V, Zn) in black shales have been used previously as paleoredox indicators of the water column (Algeo et al., 2006; Tribovillard et al., 2006; Algeo et al., 2009; Algeo et al., 2012; Meyer et al., 2008; Gordon et al., 2009; Sahoo et al., 2012; Sahoo et al., 2016). Also, they have been used to track atmospheric oxygenation through time (Scott et al., 2008; Partin et al., 2013; Lyons et al., 2014). Recently, Large et al. (2014) proposed that trace element concentrations in sedimentary pyrite formed in marine black shales could be used as proxies for ocean trace element chemistry and atmospheric oxygenation. This proxy relies on the fact that most redox-sensitive trace elements, in bottom waters and pore waters, are readily and efficiently adsorbed by sedimentary pyrites (Huerta-Diaz and Morse, 1992; Morse and Arakaki, 1993, Rickard et al., 2012; Gregory et al., 2014; Large et al., 2014; Gregory et al., 2015, 2016; Mukherjee and Large, 2016). The premise on which the technique is based, is that enhanced oxidative weathering on land causes an increase in the supply of redox-sensitive trace elements in the riverine flux (dominant source) in the ocean (Bertine et al., 1973; Taylor et al., 1995; Scott et al., 2008; Sahoo et al., 2012; Crowe et al., 2013). On encountering a redox-boundary, these become readily adsorbed by sedimentary pyrites forming in anoxic black shales. Hence, redox sensitive trace element concentrations in sedimentary pyrites act as an indirect proxy for atmospheric oxygenation events (Gregory et al., 2014; Large et al., 2014; Gregory et al., 2015a; Gregory et al., 2015; Mukherjee and Large, 2016) including the proof of concept paper (Large et al., 2014).

There are several advantages of using a marine pyrite trace element approach for understanding ocean chemistry. Firstly, the technique (LA-ICP-MS) is highly sensitive and allow in situ analysis of the pyrites. Better detection limits by LA-ICP-MS (particularly for elements like Se, Co and Mo) allow more robust trace element concentrations at lower levels. Secondly, both techniques offer high

spatial resolution so measurements can be performed within a single grain domain, avoiding the problem of variation in composition of shales. Many TE, such as Mo, are partitioned between minerals in the shale (Tribovillard et al., 2006), and thus variation in mineral composition affects the bulk rock analyses. Thirdly and most importantly, the effects of diagenesis, metamorphism and hydrothermal activity, all of which affect trace element concentrations, can easily be detected by textural study of the pyrite before and after analyses. In case of trace elements, Large et al. (2009) demonstrated that the process of recrystallization of pyrite during diagenesis or metamorphism, releases most trace elements and results in a subhedral to euhedral form of pyrite with low trace element abundances. Hydrothermal pyrites, on the other hand, may be enriched/depleted in trace elements depending on the conditions of formation (temperature, salinity and proximity to vents). These concentrations therefore do not reflect primary trace element concentrations of the sea water (Large et al., 2014; Mukherjee and Large, 2017).

1.2 Sample details

Marine, least metamorphosed, undeformed organic matter rich-sedimentary black shales aged between 500-2500 Ma, were chosen for the study from various sedimentary basins around the world (Table S1a, b). Samples were mainly collected in the form of drill cores in order to ensure the pyrite was well preserved and not oxidised. For all the drill holes, rock specimens were collected every ~10 m down-hole (approximately 20-30 samples per drill hole). From each interval of interest, rock specimens were set in 2.5 cm diameter epoxy molds and polished with 1-micron diamond paste. Polished laser mounts were prepared for petrological analysis using reflected light microscope followed by LA-ICP-MS pyrite analyses for trace elements at CODES, University of Tasmania.

Polished mounts were studied under reflected light in order to select samples that contain fine grained early-formed sedimentary pyrite and discard coarse, recrystallised and diagentically altered pyrite. Most samples comprised sedimentary pyrite in the form of individual microcrystals, aggregates of microcrystals, framboids and nodular concretions in black shales. Coarser euhedral pyrites are present in some samples, that were not analysed as their

TE budget is affected because of recrystallization (Large et al., 2014; Gregory et al., 2015; Mukherjee and Large, 2016).

1.3 LA-ICP-MS analyses of pyrite data processing

Analyses were carried out using a New Wave Research UP-193ss laser microprobe coupled to an Agilent 7700s quadrupole ICP-MS for the following elements and their respective isotopes, ^{13}C, ^{23}Na, ^{24}Mg, ^{27}Al, ^{29}Si, ^{34}S, ^{39}K, ^{43}Ca, ^{49}Ti, ^{51}V, ^{53}Cr, ^{55}Mn, ^{57}Fe, ^{59}Co, ^{60}Ni, ^{65}Cu, ^{66}Zn, ^{75}As, ^{77}Se, ^{85}Rb, ^{88}Sr, ^{90}Zr, ^{95}Mo, ^{107}Ag, ^{111}Cd, ^{118}Sn, ^{121}Sb, ^{125}Te, ^{137}Ba, ^{157}Gd, ^{178}Hf, ^{181}Ta, ^{182}W, ^{195}Pt, ^{197}Au, ^{202}Hg, ^{205}Tl, ^{206}Pb, ^{207}Pb, ^{208}Pb, ^{209}Bi, ^{232}Th and ^{238}U. STGL2b2 (in-house standard for primary calibration; Danyushevsky et al., 2011), GSD-1G (USGS reference material; Jochum et al., 2005) and a pure stoichiometric pyrite crystal (Gilbert et al., 2014) were the three primary reference materials used for the analyses; viz. quantifying siderophile and chalcophile elements, lithophile elements and sulphur abundances, respectively. The standards were analysed before unknowns and thereafter every two samples (~every 1.5 hours) as well as at the end of each run, in order to measure any analytical drift. Backgrounds were analysed for 30-seconds before the signal from the ablated sample was acquired for 40-60 seconds. The laser instrument operated with ~3.5 J/cm^2 laser fluence and 5 Hz laser repetition rate. Samples were ablated in an atmosphere of pure He flowing at a rate of 0.8 l/min, immediately after which He carrier gas was mixed with Ar (0.85 l/min) for improved efficiency of aerosol transport within the cell.

Both ICP-MS instruments were optimized to maximize sensitivity on mid to high-mass isotopes (in the range 80– 240 amu) and production of molecular oxide species (i.e., $^{232}Th^{16}O^+/^{232}Th^+$) and doubly charged ion species (i.e., $^{140}Ce^{++}/^{140}Ce^+$) were maintained at levels below 0.2%. Dwell times on each mass varied between 5 and 30 msec, depending on the count rates and total sweep time (time required to measure all isotopes once) was 0.76 sec (similar to Large et al., 2014). A total of ~10 or more spot analyses, with spot size ranging from 15-35 μm, of sedimentary pyrites were performed on each sample. Also, 5 spot analyses of black shales (material surrounding the pyrites) on each sample were carried out to measure composition of silicate matrix in the black shales. Matrix analyses were also used in the data reduction process in

order to account for matrix contamination during LA-ICP-MS analyses of pyrite grains.

The data were processed by data reduction software that uses a linear regression based algorithm for determining chalcophile and siderophile abundances relative to sulphur, for calculation of sulfide composition. The conversion of raw data (counts per seconds) into concentrations in ppm involved splitting the integration curve (curve obtained from counts per second vs analysis time) into five segments of equal duration. Each of these segments was calculated using time-equivalent calibration standards. The data (in counts per seconds) were then converted to preliminary ppm values according to standard methods (Longerich et al., 1996), using Fe as the internal standard element. The method assumes a constant stoichiometric Fe content of pyrite for calculating preliminary compositions which are then normalized to a 100% total of siderophile, chalocophile and oxides of lithophile elements. To calculate the final concentrations, a linear regression equation using S content was employed such that the sum of the chalcophile and siderophile elements including S was 100%. Additionally, regression fits for individual analyses was visually inspected. The resulting compositions were close to major element composition of stoichiometric pyrite. The precision of the analyses was calculated using regression analysis. The errors vary from analysis to analysis, depending on quality of regression fit, concentration of element, abundance of measured isotope etc. Elements such as Se, As, Sb, Ni, Zn, Cu, Pb and Co at high concentrations, relative error is under 20% for 75% of analyses. However, uncertainty increases at lower concentrations. The analytical errors sourced from the uncertainty on composition of the reference materials is estimated under 5% (Danyushevsky et al., 2011). Considering that variations between grains in a sample typically is about 100% and concentration of an element can span 2-3 orders of magnitude different samples, we conclude that observed trends are outside of analytical uncertainty.

The analytical procedure was corrected for the laser-induced element fractionation of elements with different volatilities by the application of three

calibration standards: pure pyrite for control of Fe-S fractionation; GSD-2 glass for lithophile elements; and, in-house Li-B glass STGL3 for chalcophile elements. The correction for down-hole fractionation was achieved by splitting the analyses into five segments of equal duration and calculation of elemental concentrations using time-equivalent calibration standards.

Variations in drill rate and ablation yield were also taken into account. Pyrite has substantially higher ablation yield then silicate minerals. However, the normalisation of the analyses in total results in estimation of the proportion of pyrite and sedimentary matrix in the mixed signal and hence provided a correction for this effect. Another consequence of difference in ablation yield of pyrite and sedimentary matrix is that analyses of matrix have substantially higher detection limits than the analyses of pyrite. This effect was mitigated by measurement of matrix composition with larger spot size (≈50 μm). The instrumental sensitivity drift was tracked by the measurements of the calibration standards before and after the analyses and by the application of linear corrections to the analyses. Typically, drift in instrumental sensitivity was below 5%. Precision was calculated using regression analysis. The uncertainties in values were quantified as 90% confidence intervals, which were obtained from the regression assuming identical errors on the measurements.

1.4 Se and Se/Co in pyrite as oxygenation proxies

Trace elements such as Mo, Zn, Se, Pb, Co, Cu, Bi, As are known to be redox-sensitive (i.e. multiple valence states), and respond to changes in atmosphere-ocean redox conditions (Calvert and Pedersen, 1993; Jones and Manning, 1994; Wignall, 1994; Crusius et al., 1996; Dean et al., 1997, 1999; Yarincik et al., 2000; Morford et al., 2001; Pailler et al., 2002). Oxidative weathering results in the oxidized form of these trace elements, while reduced forms occur on encountering the redox boundary in the water column or sediment-water interface where they form complexes with organic acids and become incorporated into authigenic sulphides. Not all redox sensitive elements in pyrite can be used to infer atmospheric redox conditions because of partitioning into different phases (organic, detrital) other than pyrite. For instance, Ni and Cu may be adsorbed onto organic complexes and Fe-Mn oxides/hydroxides in the sedimentary process. Silver and Tl are not redox sensitive; however, Tl in pyrite

is can be used to screen pyrites with hydrothermal effects (Mukherjee and Large, 2016). Some redox sensitive elements (Pb, As) in pyrites may not record any oxygenation trend solely due to their abundant supply in the water column (high source flux). Arsenic is particularly problematic due it being relatively more mobile under reducing conditions than other redox sensitive TE (Smedley and Kinniburgh, 2002).

An increase in redox sensitive elements such as Se suggests a possible increased supply of these elements into the water column via oxidative weathering on land. Decrease in concentrations of elements such as Co and Bi can be used to support the oxidative weathering process on land because these elements tend to be retained by Fe-Mn hydroxides and oxides during oxidative weathering (Pickering, 1979; Jackson, 1998; Huang and Germida, 2002; Sparks, 2003; Violante et al., 2008). Cobalt, owing to its cationic speciation, is strongly retained by ferro-manganese hydroxides and more so by manganese oxides and its supply to the ocean is inhibited by oxidative processes on land (Pickering, 1979; Jackson, 1998; Huang and Germida, 2002; Sparks, 2003; Violante et al., 2008). Thermodynamic modelling of the concentration of Co in the oceans (Zerkle et al., 2005) as well Co in sedimentary pyrite (Large et al., 2015) and Co/Ti in iron formations (Swanner et al., 2014) confirm this behavior where a decrease in Co concentrations through time has been observed. The element Bi enters the marine realm via two main sources; atmospheric inputs i.e., eolian dust of volcanic origin (Lee et al., 1985; 1986) or river influx, both being comparable as source material (Bertine et al., 1996). Bismuth, owing to its extensive hydrolytic activity and strong particle reactivity, is also retained in oxyhydroxides, particularly manganese phases (Barnes, 1967; Fowler et al., 2010). Currently there is no thermodynamically modelled trend for Bi in the oceans through time. Nevertheless, Large et al. (2014) pointed out that Bi was one of the least abundant trace elements in the ocean today with a very short residence time (on the order of 20 years) and, like Co, likely decreased in concentration with increasing atmosphere oxygenation.

We propose that the use ratios of two elements that exhibit an antithetic behavior i.e., one element commonly increases in concentration with atmosphere oxidation (e.g., Se, Zn, Mo, Ni), and the other decreases in

concentration (e.g., Co, Bi) is a more robust way to evaluate atmosphere oxygenation.

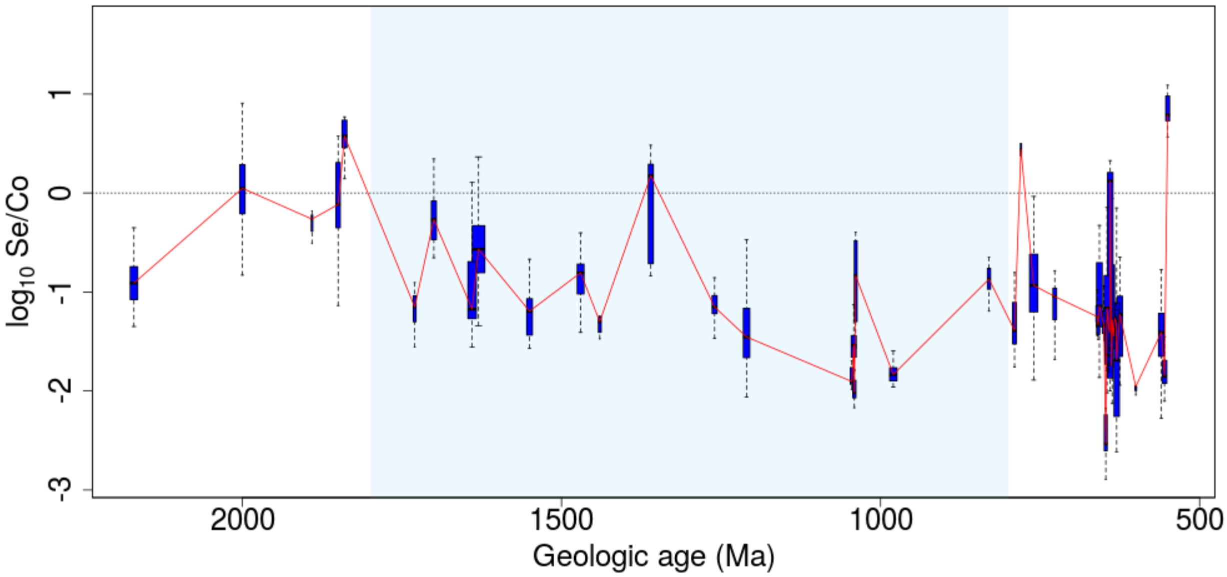

Fig SI 1 Plot of the log-ratio of Se to Co against geologic age. Shown are boxplots for each sample. The median trend is shown as a solid line.

References

Algeo T.J., Lyons T.W., 2006. Mo–total organic carbon covariation in modern anoxic marine environments: Implications for analysis of paleoredox and paleohydrographic conditions. Paleoceanography 21, 1- 23.

Algeo T.J., Rowe H., 2012. Paleoceanographic applications of trace-metal concentration data. Chemical Geology 324, 6–18.

Algeo T.J., Tribovillard N., 2009. Environmental analysis of paleoceanographic systems based on molybdenum-uranium covariation. Chemical Geology 268, 211–225.

Barnes S.S., 1967. Minor element composition of ferromanganese nodules. Science 157, 63-65.

Bertine K. K., Koide M., and Goldberg E.D., 1996. Comparative marine chemistries of some trivalent metals-bismuth, rhodium and rare Earth elements. Mar. Chem. 53, 89–100.

Bertine, K.K., Turekian, K.K., 1973. Molybdenum in marine deposits. Geochim. Cosmochim. Acta 37, 1415–1434.

Calvert, S.E., Pedersen, T.F., 1993. Geochemistry of Recent oxic and anoxic marine sediments: implications for the geological record. Mar. Geol. 113, 67–88.

Crowe, S.A., Døssing, L.N., Beukes, N.J., Bau, M., Kruger, S.J., Frei, R., Canfield, D.E., 2013. Atmospheric oxygenation three billion years ago. Nature 501, 535–538.

Crusius, J., Calvert, S., Pedersen, T., Sage, D., 1996. Rhenium and molybdenum enrichments in sediments as indicators of oxic, suboxic and sulfidic conditions of deposition. Earth Planet. Sci. Lett. 145, 65–78.

Danyushevsky, L., Robinson, P., Gilbert, S., Norman, M., Large, R., McGoldrick, P., Shelley, M., 2011. Routine quantitative multi-element analysis of sulphide minerals by laser ablation ICP-MS: Standard development and consideration of matrix effects. Geochem., Explor. Environ. Anal. 11, 51–60.

Dean, W.E., Gardner, J.V., Piper, D.Z., 1997. Inorganic geochemical indicators of glacial – interglacial changes in productivity and anoxia of the California continental margin. Geochim. Cosmochim. Acta 61, 4507– 4518.

Dean, W.E., Piper, D.Z., Peterson, L.C., 1999. Molybdenum accumulation in Cariaco basin sediment over the past 24 k.y.: a record of water-column anoxia and climate. Geology 27, 507– 510.

Fowler Scott W., Teyssie Jean-Louis, Church Thomas M., 2010. Scavenging and retention of bismuth by marine plankton and biogenic particles, Limnology and Oceanography 55, 1093–1104.

Gilbert S., Danyushevsky L., Goemann K. and Death D. (2014a) Fractionation of sulphur relative to iron during laser ablation-ICP-MS analyses of sulphide minerals: implications for quantification. J. Anal. At. Spectrom. 29, 1024–1033.

Gordon, G.W., Lyons, T.W., Arnold, G.L., Roe, J., Sageman, B.B., Anbar, A.D., 2009. When do black shales tell molybdenum isotope tales? Geology 37, 535–538.

Gregory, D., Meffre, S., and Large, R., 2014, Comparison of metal enrichment in pyrite framboids from a metal-enriched and metal-poor estuary: American Mineralogist, v. 99, no. 4, p. 633-644.

Gregory, D.D., Large, R.R., Halpin, J.A., Lounejeva Baturina, E., Lyons, T.W., Wu, S., Sack, P.J., Chappaz, A., Maslennikov, V.V., Bull, S.W., Danyushevsky, L., 2015. Trace element content of sedimentary pyrite in black shales. Econ. Geol. 110, 1389–1410.

Huang, P.M., and Germida, J.J., 2002. Chemical and biochemical processes in the rhizosphere: metal pollutants. In Interactions Between Soil Particles and Microorganisms: Impact on the Terrestrial Ecosystem, ed. Huang, P. M., Bollag, J.M., and Senesi, N., Wiley, New York, 381–438.

Huerta-Diaz, M.A., Morse, J.W., 1992. Pyritization of trace metals in anoxic marine sediments. Geochim. Cosmochim. Acta 56, 2681–2702

Jackson, T.A., 1998. The biogeochemical and ecological significance of interactions between colloidal minerals and trace elements. In Environmental Interactions of Clays, ed. Parker, A., and Rae, J. E., Springer-Verlag, Berlin, 93–205.

Jochum, K.P., Pfänder, J., Woodhead, J.D., Willbold, M., Stoll, B., Herwig, K., Amini, M., Abouchami, W. and Hofmann, A.W., 2005. MPI-DING glasses: New geological reference materials for in situ Pb isotope analysis. Geochemistry Geophysics Geosystems 6, 1525-2027.

Jones, B., Manning, D.A.C., 1994. Comparison of geochemical indices used for the interpretation of palaeoredox conditions in ancient mudstones. Chem. Geol. 111, 111 – 129.

Large, R.R, Halpin, J.A., Lounejeva, E., Danyushevsky, L.V., Maslennikov, V.V., Gregory, D., Sack, P.J., Haines, P.W., Long, J.A., Makoundi, C., Stepanov, A.S., 2015. Cycles of nutrient trace elements in the Phanerozoic ocean, Gondwana Research 28 (4), 1282-1293.

Large, R.R., Danyushevsky, L., Hollit, C., Maslennikov, V., Meffre, S., Gilbert, S., Bull, S., Scott, R., Emsbo, P., Thomas, H., Singh, B., Foster, J., 2009. Gold and trace element zonation in pyrite using a laser imaging technique: Implications for the timing of gold in orogenic and carlin-style sediment-hosted deposits. Econ. Geol. 104, 635–668.

Large, R.R., Halpin, J.A., Danyushevsky, L.V., Maslennikov, V.V., Bull, S.W., Long, J.A., Gregory, D.D., Lounejeva, E., Lyons, T.W., Sack, P.J., McGoldrick, P. and Calver, C.R., 2014. Trace element content of sedimentary pyrite as a new proxy for deep-time ocean- atmosphere evolution. Earth and Planetary Science Letters 389, 209-220.

Lee, D.S., Edmond, J.M., and Bruland, K.W., 1985/1986. Bismuth in the Atlantic and North Pacific Ocean: A natural analogue to plutonium and lead? Earth Planet. Sci. Lett. 76, 254–262.

Longerich, H.P., Jackson, S.E. and Günther, D., 1996. Laser ablation inductively coupled plasma mass spectrometric transient signal data acquisition and analyte concentration calculation. J. Anal. At. Spectrom. 11, 899-904.

Lyons, T.W., Reinhard, C.T., Planavsky, N.J., 2014. The rise of oxygen in Earth's early ocean and atmosphere. Nature 506, 307-315.

Meyer KM, Kump LR. 2008. Oceanic euxinia in Earth history: Causes and consequences. Annu. Rev. Earth Planet. Sci. 36, 251–88.

Morford, J.L., Russell, A.D., Emerson, S., 2001. Trace metal evidence for changes in the redox environment associated with the transition from terrigenous clay to diatomaceous sediment, Saanlich Inlet, BC. Mar. Geol. 174, 355– 369.

Morse, J.W., Arakaki, T., 1993. Adsorption and coprecipitation of divalent metals with mackinawite (FeS). Geochim. Cosmochim. Acta 57, 3635–3640

Mukherjee, I., and Large, R., 2016. Pyrite trace element chemistry of the Velkerri Formation, Roper Group, McArthur Basin: Evidence for atmospheric oxygenation during the Boring Billion: Precambrian Res. 281, 13-26

Mukherjee, I., and Large, R., 2017. Application of pyrite trace element chemistry to exploration for SEDEX style Zn-Pb deposits: McArthur Basin, Northern Territory, Australia. Ore Geology Reviews 81, 1249-1270.

Pailler, D., Bard, E., Rostek, F., Zheng, Y., Mortlock, R., van Geen, A., 2002. Burial of redox-sensitive metals and organic matter in the equatorial Indian Ocean linked to precession. Geochim. Cosmochim. Acta 66, 849– 865.

Partin, C.A., Bekker, A., Planavsky, N.J., Scott, C.T., Gill, B.C., Li, C., Podkovyrov, V., Maslov, A., Konhauser, K.O., Lalonde, S.V., Love, G.D., Poulton, S.W., Lyons, T.W., 2013. Large-scale fluctuations in Precambrian atmospheric and oceanic oxygen levels from the record of U in shales. Earth Planet. Sci. Lett. 369–370.

Pickering, W., 1979. Copper retention by sediment/soil components. In Copper in the Environment Vol. I, Ecological Cycling, ed. Nriagu, J. O., Wiley, New York, 217–253

Rickard, D., 2012. Sulfidic sediments and sedimentary rocks. In: Van Loon, A.J. (Ed.), Developments in Sedimentology. Elsevier, p. 801.

Sahoo, S. K., Planavsky, N. J., Jiang, G., Kendall, B., Owens, J. D., Wang, X., Shi, X., Anbar, A. D. and Lyons, T. W., 2016. Oceanic oxygenation events in the anoxic Ediacaran ocean. Geobiology 14, 457–468.

Sahoo, S.K., Planavsky, N.J., Kendall, B., Wang, X., Shi, X., Scott, C., Anbar, A.D., Lyons, T.W., Jiang, G., 2012. Ocean oxygenation in the wake of the Marinoan glaciation. Nature 489, 546–549.

Scott, C., Lyons, T.W., Bekker, A., Shen, Y., Poulton, S.W., Chu, X., Anbar, A.D., 2008. Tracing the stepwise oxygenation of the Proterozoic ocean. Nature 452, 456–459.

Smedley, P L, and Kinniburgh, D G. 2002. A review of the source, behaviour and distribution of arsenic in natural waters. Applied Geochemistry 17, 517–568

Sparks, D.L., 2003. Environmental Soil Chemistry, 2nd ed. Academic Press, San Diego, CA. pp.352

Swanner, E.D., Planavsky, N.J., Lalonde, S.V., Robbins, L.J., Bekker, A., Rouxel, O.J., et al., 2014. Cobalt and marine redox evolution. Earth Planet. Sci. Lett. 390, 253–263.

Taylor, S.R., Mclennan, S.M., 1995. The geochemical evolution of the continental crust. Rev. Geophys. 33, 241–265.

Tribovillard, N., Algeo, T.J., Lyons, T., Riboulleau, A., 2006. Trace metals as paleoredox and paleoproductivity proxies: an update. Chem. Geol. 232, 12–32.

Violante, A., Krishnamurti, G.S.R., Pigna, M. 2008. Mobility of Trace Elements in Soil Environments. In: A. Violante, P.M. Huang, G.M. Gadd (eds). Biophysico-Chemical Processes of Metals and Metalloids in Soil Environments. John Wiley and Sons, Hoboken, NJ, pp.169-213.

Wignall, P.B., 1994. Black Shales. Clarendon Press, Oxford. 127 pp.

Yarincik, K.M., Murray, R.W., Lyons, T.W., Peterson, L.C., Haug, G.H., 2000. Oxygenation history of bottom waters in the Cariaco Basin, Venezuela, over the past 578,000 years: results from redox-sensitive metals (Mo, V, Mn, and Fe). Paleoceanography 15, 593– 604

Zerkle, A.L., House, C.H., Brantley, S.L., 2005. Biogeochemical signatures through time as inferred from whole microbial genomes. Am. J. Sci. 305, 467–502.
